# Findings from the ‘Ask Me About PrEP’ HIV Pre‐Exposure Prophylaxis Awareness Programme in England

**DOI:** 10.1111/jep.14163

**Published:** 2024-10-23

**Authors:** Jonny Edwards, Sara Paparini, Will Nutland, Marc Thompson, Phil Samba

**Affiliations:** ^1^ University of Lincoln Lincoln UK; ^2^ SHARE Collaborative, Wolfson Institute of Population Health Queen Mary University of London London UK; ^3^ The Love Tank CIC London UK

**Keywords:** awareness, HIV, intervention, peer, Pre‐Exposure Prophylaxis, PrEP

## Abstract

**Rationale:**

HIV incidence has decreased in England by over a third since 2019. Despite the early success of HIV Pre‐Exposure Prophylaxis (PrEP) in reducing HIV transmission in the United Kingdom, many people who could benefit from HIV PrEP do not yet know about it, or know how to access it.

**Aims and Objectives:**

This paper presents the findings of the first England‐wide national peer‐to‐peer based diffusion model to disseminate information about PrEP. Ask Me About PrEP (AMAP) was a 5‐month pilot programme which commenced in November 2021. Volunteer mobilisers were encouraged to use their existing knowledge of their communities and geographical areas to assist them in discussing PrEP with their peers.

**Method:**

12 enrolled mobilisers took part in three project evaluation focus groups between November 2021 and March 2022, and five AMAP project staff took part in one focus group in March 2022. Additionally, descriptive statistical analysis explored volunteer mobiliser recruitment to the AMAP project, demographical data of project staff and volunteer mobilisers, volunteer mobiliser attrition rates, and the project's impact.

**Results:**

96 volunteers enrolled, completed training and volunteered as mobilisers. Thoroughout the project, mobilisers engaged their peers in 11,889 conversations about PrEP through individual conversations, online group conversations, online workplace educational events, and social media. The focus groups enabled key stakeholders to reflect on their experiences of the pilot programme. Four key themes were identified during the focus groups: motivations to mobilise and recruitment experiences; training, learning, and materials; mobilisation activity; and support and social networking.

**Conclusion:**

Our evaluation demonstrates that peer‐to‐peer diffusion models used to increase awareness of HIV PrEP in key unreached groups, offer an acceptable public health intervention model for volunteers and project staff.

## 1 INTRODUCTION

HIV incidence has decreased in England by over a third since 2019, placing the country on track towards achieving its goal of ending new HIV transmission by the year 2030.^1^ The downward trajectory of HIV diagnoses is largely attributed to increased uptake of HIV testing and the roll‐out of HIV Pre‐Exposure Prophylaxis (PrEP), an HIV prevention medication, on the National Health Service in Autumn 2020.^2^ Despite PrEP's contribution to reducing HIV incidence, many people who could benefit from HIV PrEP do not yet know about it, or how to access it. Young gay men (aged 16–24 years), trans people, cisgender women, people at risk of HIV through heterosexual sex (including Black African and Black Caribbean people) and people from a low socioeconomic background, are less likely to know about PrEP and are less likely to access it.^3^

Existing literature demonstrates peer‐based approaches to PrEP education in varying forms are effective, highly acceptable, and often preferable to non‐peer‐based approaches especially when PrEP users themselves provide information to potential PrEP beneficiaries.^4^, ^5^, ^6^, ^7^, ^8^, ^9^, ^10^, ^11^, ^12^, ^13^, ^14^, ^15^, ^16^, ^17^, ^18^, ^19^ Different models of PrEP peer‐to‐peer diffusion have been evaluated in the United States, West African countries, Kenya, the Dominican Republic, Tanzania, Zambia, Nigeria, Uganda, and South Africa.^4^, ^5^, ^6^, ^7^, ^8^, ^9^, ^10^, ^11^, ^12^, ^13^, ^14^, ^15^, ^16^, ^17^, ^18^, ^19^ However, such models have not yet been formally evaluated before as a national intervention in England, with the exception of PrEPster's initial MobPrESH programme,^20^ which was a regional intervention led by PrEPster in association with two partner organisations in Yorkshire, and Bristol. This paper thus provides the first evaluation evidence of an England‐wide national peer‐based model.

### 1.1 Intervention design and delivery

Ask Me About PrEP (AMAP) was a 5‐month pilot programme, funded through the HIV and Sexual and Reproductive Health Innovation Fund (formerly administered by Public Health England, now UK Health Security Agency), which commenced in November 2021.

AMAP was underpinned by a combination of theoretical models including the Diffusion of Innovation Theory^21^ (which explains how health behaviours such as PrEP use are adopted by a community), and Social Network Theory^22^ (which use existing networks to influence behaviour change through modelling and persuasion, such as through conversations). Additionally, AMAP design was informed by Public Health England's’ Guide to Community‐Centred Approaches.^23^

**Figure 1:**
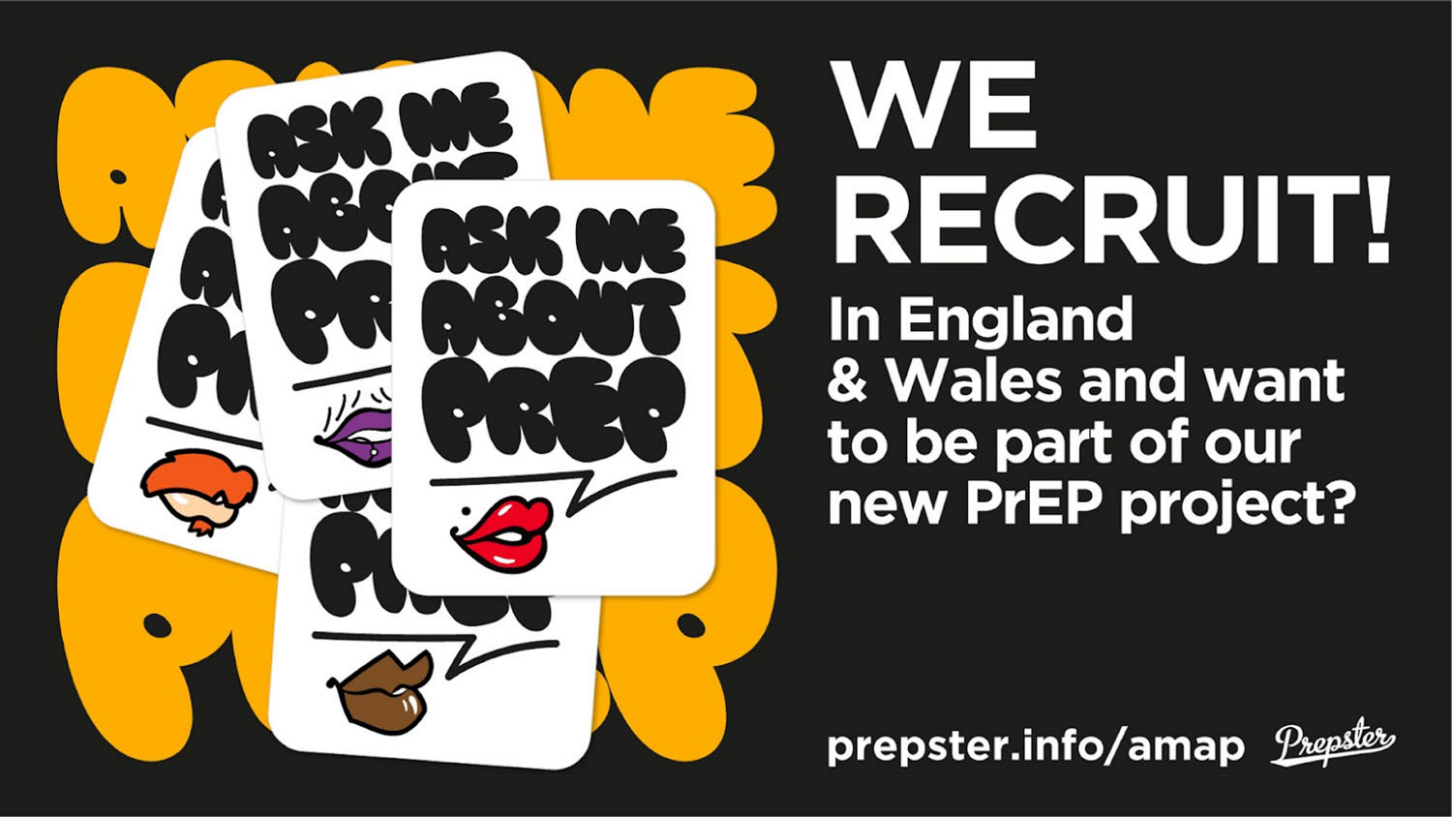
Social Media Recruitment Post.

96 volunteer mobilisers completed the required training and volunteered as mobilisers during the pilot programme in England. Mobilisers were recruited via social media posts (see Figure 1), through information cards (distributed via venues, networks, and partner organisations), and word of mouth. A link to a website page was provided which outlined what participation in the pilot programme would involve (https://prepster.info/amap/). Potential mobilisers were asked to complete a brief online enrolment form, including sections that asked them to describe their motivations for wanting to take part in AMAP, and how they might engage with their peers about PrEP. To assist in triaging potential mobilisers, participants were asked to indicate which region of England they lived in, and to provide their age.

**Figure 2:**
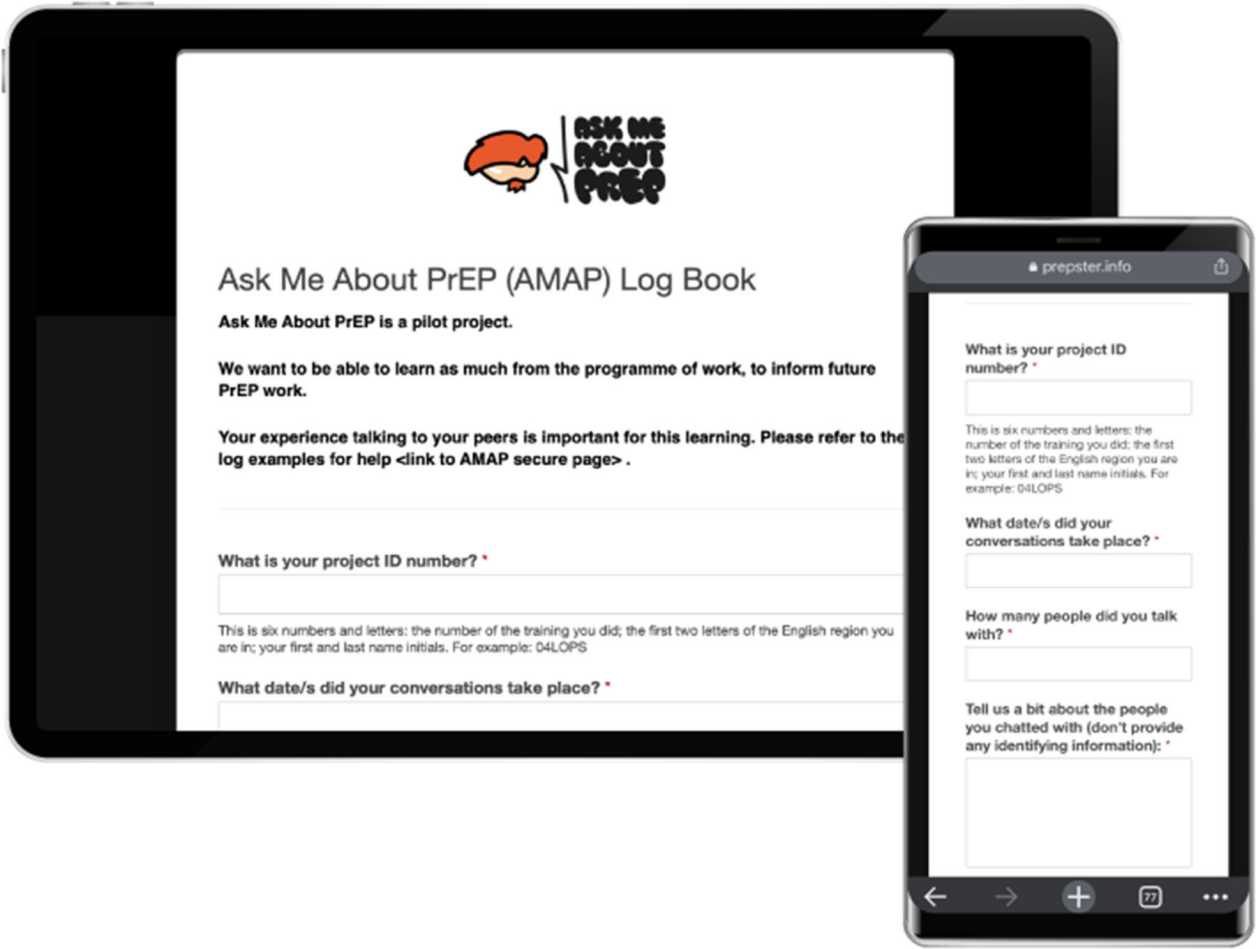
AMAP Log Book (used by mobilisers to record activity).

**Figure 3:**
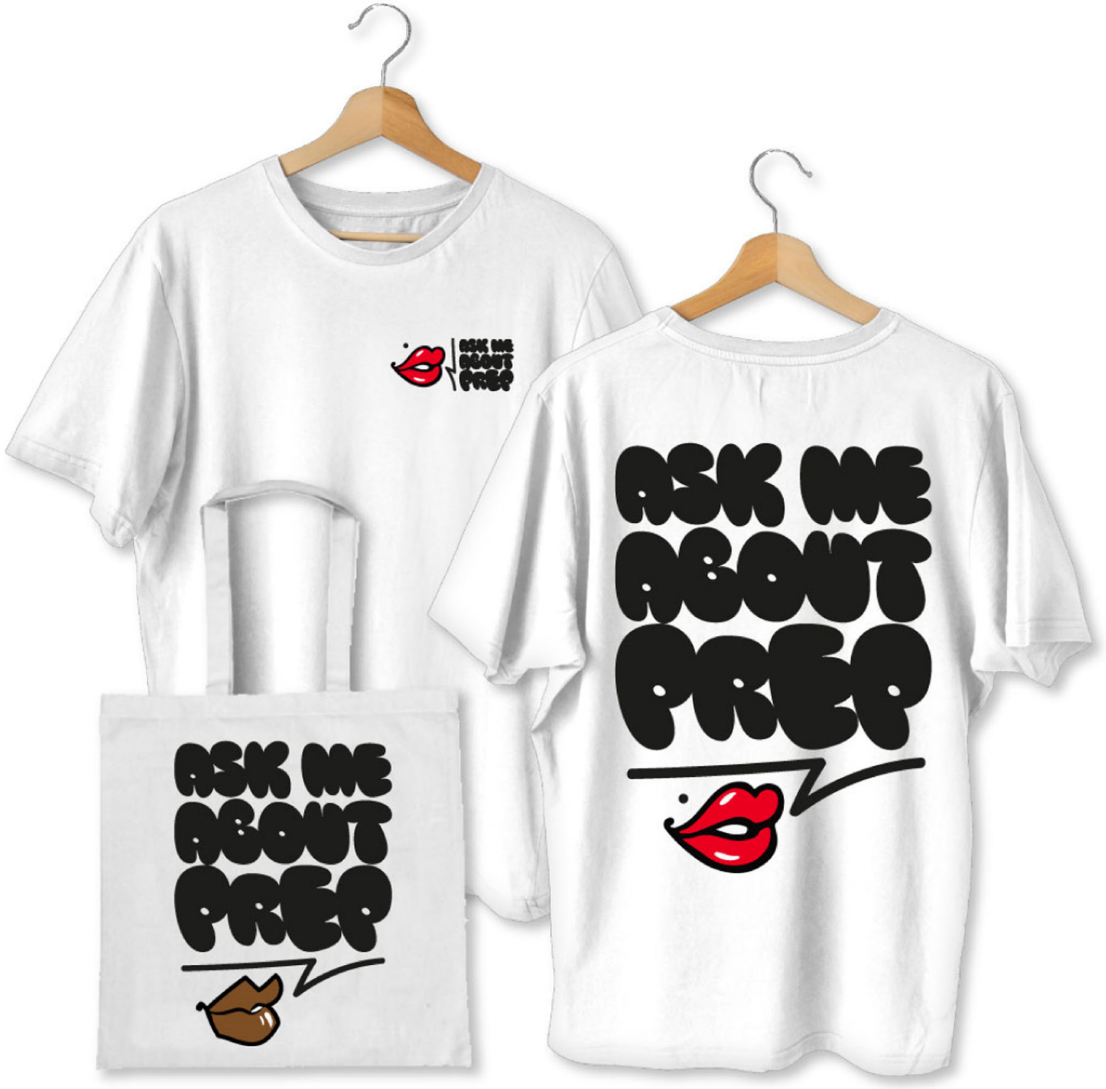
AMAP Merchandise (examples of free merchandise available to mobilisers).

Overall, twenty four online training events took place, with two additional in‐person events. One event was held in partnership with the community organisation George House Trust, in Manchester. Another event was delivered for an adult entertainment business, in London. Mobiliser training content was developed by members of the PrEPster team and continually reviewed and adapted in response to feedback. Mobilisers were provided with a set of ‘top‐six’ PrEP information resources, including sources of information that would assist them in deepening their personal knowledge of PrEP. During the training events, mobilisers were shown how to access and complete monitoring and activity reporting (log book) forms (see Figure 2), which were designed to enable ongoing project impact evaluation. Mobilisers were also instructed on how to access an online platform, to order free promotional materials such as tee‐shirts and printed booklets (see Figure 3), to support them in their mobilisation activities.

Mobilisers were encouraged to use their existing knowledge of their communities to assist them in discussing PrEP with their peers, in ways that felt most comfortable to them. This included posting about PrEP on social media, having individual or group conversations with peers (in person and/or online), and hosting PrEP specific information events. Enrolled mobilisers were kept up to date with the progress of the programme, with regular emails and invitations to attend online support meetings with other mobilisers. Mobilisers were also provided with the contact details of a member of the project team who acted as a mentor, should the mobilisers require any additional one‐to‐one support.

Our evaluation study—reported on below—seeks to assess the project and reflect upon outcomes and key learning points arising from the implementation of AMAP as the first England‐wide national peer diffusion PrEP information programme.

## 2 MATERIALS AND METHODS

This paper reports on two sets of data, collected during our evaluation. The first set of data (Project Performance and Impact) is a descriptive statistical analysis which explores volunteer mobiliser recruitment to the AMAP project, demographical data of project staff and volunteer mobilisers, and volunteer mobiliser attrition rates. Analysis explored the project impact data (volunteer mobiliser reported activities via online log book data), including types of interventions and reported outcomes of interventions that were undertaken by the volunteer mobilisers, alongside a demographical analysis of the beneficiaries of the interventions. The data which forms this analysis was collected by PrEPster throughout the duration of the project. WN collated the anonymised data, which was then provided to JE, who utilised Excel to summarise and organise the data before presenting the data in textual format.^24^ JE is a practicing clinician with a background in primary care/general practice and is a PhD student exploring the impact of activist organisation‐led HIV interventions and services in England. JE is also a PrEP user and gay man.

The second set of data (Stakeholder Experiences) is a qualitative enquiry into the experiences of key stakeholders taking part in the AMAP pilot project, through a series of qualitative project evaluation focus groups. The qualitative enquiry was undertaken by two independent researchers (JE and SP). SP is a senior lecturer and experienced qualitative researcher with expertise in HIV and PrEP research.

Recruitment information for the evaluation was emailed to potential evaluation participants, asking them to contact the researchers should they wish to take part in the study. Three mobiliser focus groups were held between December 2021 and March 2022. One additional focus group with project staff was held in March 2022. Focus groups were held on‐line via Microsoft Teams or Zoom. In total, four focus groups were recorded and later manually transcribed by JE. Data was coded and analysed using thematic analysis.^25^

Topic guides were developed before focus groups to provide a semi‐structured, flexible approach to discussions. Seven pre‐prepared questions (see Appendix A) were presented to the enrolled volunteer mobiliser focus group participants. Additionally, nine pre‐prepared questions were asked to members of staff (See Appendix B) to gather information on their experiences of the project.

All participants received an information sheet detailing the process and aims of the study. The participants consented to taking part in the study and to the recording of the focus groups. A recorded verbal consent method was chosen to increase accessibility for participants because of the potential for variable levels of literacy and language ability and the potential concerns apropros power dynamics and possible risk of community suspicion surrounding the perceived officialdom of signing research consent forms.^26^ All participants consented to their information being used in evaluation dissemination activities such as publications, reports, and conferences. Participants were advised that they could freely withdraw from the evaluation at any point and that their information would not be used in the study.

As an evaluation, this project did not require ethical approval but adhered to ethical principles of practice in the UK, such as the Declaration of Helsinki.^27^, ^28^ This enabled the team to respect participants’ rights, delivering the evaluation with respect and dignity for those involved. Data was stored and processed in accordance with The Data Protection Act.^29^

## 3 RESULTS—PROJECT PERFORMANCE AND IMPACT

### 3.1 Mobiliser recruitment

138 people registered to attend online training and join the programme via the website. 58 people (42%) did not go on to attend online training (pre‐training attrition). In total 80 people who registered interest completed online training. Additionally, a further 9 people registered for and attended face‐to‐face training provided in Manchester and 7 people attended face‐to‐face training provided in London. There were no people who had registered to attend face‐to‐face training that did not complete training. In total 16 people undertook face‐to‐face training. Overall, 96 people completed training.

### 3.2 Demographic data of volunteers who completed training

The following demographic data was provided by volunteers upon completion of training. 95 out of 96 mobilisers provided demographic data for analysis. 1 mobiliser declined to provide demographic data. Volunteers self‐reported their ages: 25 years or under (19%), 26–30 years (23%), 31–35 years (21%), 36–40 years (8%), 41–45 years (12%), 46–50 years (7%), 51–55 years (2%), 56–60 years (5%), 61–65 years (2%), and 66 years or above (1%).

Volunteers self‐reported their ethnicity: White British (40%), Black & Black Mixed (19%), White Other (13%), Asian & Asian Mixed (11%), Latino (8%), White Irish (5%), Arab (2%), and Other Ethnicity (2%). 33% of volunteers were not born in the UK.

Volunteers self‐reported their combined gender/sexuality as gay male including transgender men (54%), as bisexual male including transgender men (10%), queer male (9%), heterosexual cisgendered female (6%), gay non‐binary (5%), bisexual female (5%), queer non‐binary (4%), queer female (3%), lesbian cisgendered female (2%), and bisexual non‐binary (2%). In total, 7% of volunteers reported personal experience of undertaking sex work.

### 3.3 Post‐training attrition

8 (8%) withdrew from the project in the period after training and before project completion. 7 people reported that this was due to a change in personal circumstance and 1 person reported that this was due to the programme not being as they had expected. In total, 96 volunteers completed training and volunteered as mobilisers. Accounting for 8% post‐training attrition, 88 mobilisers remained until the end of the pilot programme.

### 3.4 Log book activity impact data

Upon completion of training, mobilisers began undertaking interventions in their communities. Mobilisers returned completed log books detailing their activities to allow for continuous project impact analysis. 152 log book forms were returned, up until the end of April 2022. Over the duration of the project mobilisers enabled 11,889 conversations about PrEP. Log book forms recorded 337 one‐to‐one conversations about PrEP (3% of total conversations). 484 people engaged directly in online group conversations about PrEP (4% of total conversations) (e.g., Twitter space attendees and group WhatsApp chats) with a further 68 individuals (1% of total conversations) engaging in workplace online training events and forums about PrEP. 80 individuals (24%) reported that they were going to seek PrEP (including PrEP resumption in a small number of cases) as a direct result of one‐to‐one conversations.

Additionally, a small number of mobilisers created their own social media video content about PrEP. Two such videos generated in excess of 11,000 engagements (93% of total conversations) on Twitter or Instagram, with one of the videos viewed by 35,000 individuals. No data reported an outcome of beneficiaries seeking PrEP after online engagement was reported.

It was not always possible for mobilisers to elicit demographic data from peer beneficiaries. In many instances beneficiaries could engage anonymously, and in some instances mobilisers did not report demographic data. Self‐reported ages were provided for 152 of the people encountered: 20 years or under (5%), 21–29 years (50%), 30–39 years (22%), 40–49 years (11%), 50–59 years (7%), 60–69 years (3%), and 70 years or over (2%). Self‐reported gender was provided for 297 of the people encountered: male (67%); female (15%), non‐binary (3%) and transgender (15%).

Self‐reported ethnicity was provided for 131 of the individuals encountered: any Asian Ethnicity (22%), Gypsy or Irish Traveller (22%), White British (20%), Black (including Black African or Black Caribbean) (15%), White Other (11%), and any Other Ethnicity (10%). Self‐reported sexual identity was provided for 139 of those who mobilisers encountered: gay (75%), heterosexual (12%) bisexual (8%), and queer (4%).

Log book data reported settings in which PrEP conversations were undertaken, including through dating apps, through social media, in venues (including saunas and sex clubs), workplaces, educational settings, libraries, social groups, in the street, in bedrooms, at dinner parties, and cruising grounds.

Log book data demonstrated that sometimes the encounters were initiated and planned (e.g. a pre‐announced time on social media, a workplace event, a venue to start PrEP conversations). Frequently, encounters were unplanned, opportunistic or spontaneous (e.g. students asking a mobiliser about the AMAP sticker on their lap top, a dinner party conversation, an AMAP tee shirt worn at the gym or at a party, or the Ask Me About PrEP profile “stickers” on social media profiles). More spontaneous conversations reached people who were much less likely to have ever heard of PrEP, or to move in circles where PrEP is spoken about. Log book data also highlighted that conversations occured with people who were currently using PrEP and/or had previously done so. Those conversations explored issues including dosing regimes, side effects, issues with travel whilst carrying PrEP, drug interactions, or issues booking follow up appointments.

## 4 RESULTS—STAKEHOLDER EXPERIENCES

14 mobiliser participants were recruited in total, 12 attended, 2 did not attend. Additionally, all project coordinators (5 staff members) attended a staff specific focus group.

### 4.1 Demographic data (mobiliser participants)

Mobiliser participants self‐reported their ages: 21–29 years (8%), 30–39 years (50%), 40–49 years (17%), 50–69 years (17%), 70–89 (8%). Mobiliser participants self‐reported their ethnicity: White British (67%), Asian British (17%), Black Carribean (8%), Gypsy/Irish Traveller (8%). Mobiliser participants self‐reported their combined gender/sexuality as gay cisgendered male (83%) and gay or bisexual transgender male (17%). Participants lived in London (42%), the Midlands (17%), the South East (17%), the North West (17%), and in the North East (8%). Participants had been enrolled as mobilisers for 3 months (59%), 2 months (25%), 5 months (8%) and 1 month (8%).

### 4.2 Demographic data (staff participants)

Staff participants self‐reported their ages: 55 years (20%), 53 years (20%), 30 years (40%), and 24 years (20%). Staff participants self‐reported their gender: cisgendered male (80%) and non‐binary (20%). Staff participants self‐reported their ethinicity: White British (20%), Black British (20%), Black African (20%), Asian British (20%), and Latino (20%). All staff participants self‐reported their sexuality as queer (100%). All staff participants were London based (100%).

### 4.3 Motivations to mobilise and recruitment experiences

Participants were highly motivated to take part in the pilot project and inspired to give back to their communities by increasing the awareness and uptake of PrEP. Many participants had personal experiences of living through the early stages of the HIV epidemic and were impacted by the stigma associated with an HIV diagnosis. Some participants had experienced their own journey of receiving a diagnosis of HIV, whilst others had worked as clinicians treating people who had received an HIV diagnosis. All participants had identified specific PrEP knowledge gaps within their own communities or based on their own life experiences. Identified PrEP knowledge gaps included: a lack of information about PrEP amongst people living with mental health challenges, people with disabilities, older people (>40 years old), men who have sex with men that use saunas, and transgender people.

Mobilisers also spoke of their motivation to continue after the programme had concluded, with all participants expressing that they would continue to mobilise independently or, should the project continue, as part of the AMAP programme. This motivation was often linked to their personal experiences and original desire to take part in the project. Despite the high level of motivation experienced by participants, the AMAP team reported that they experienced some challenges in converting people who initially registered interest into active volunteers. The initial pilot aim was to recruit and train 150 mobilisers.

Initially, 154 total prospective volunteers registered interest in becoming mobilisers, however only 96 mobilisers were recruited and trained in total. Staff participants explained that setting a lower initial recruitment figure (100–120), would have been more achievable. Furthermore, staff reported that incentivisation such as the gifting of vouchers may have supported early recruitment into the project. Mobiliser recruitment strategies were highly successful in ensuring that the cohort of enrolled mobilisers was diverse, including representatives of the project's target groups ‐ people considered to be more involved in HIV acquisition risk, and simultaneously less likely to know about, or access PrEP.

### 4.4 Training, learning, and materials

The training programme was designed and delivered by AMAP staff. The training was subject to an iterative review process, which enabled the staff to continuously improve the delivery and content of training based on post‐training feedback from mobilisers. Training was developed to be ‘top‐level’ and relatively short, to be useful to busy people who could not engage with a longer training programme. General feedback from mobilisers after training was significantly positive, with participants often feeling inspired and empowered. Participants highly valued the option to watch a recording of the training at a later stage to reinforce their learning.

Some participants felt that they would have preferred face‐to‐face sessions, and interactive workshops, perhaps based on case studies and simulated exercises. However, the impact of Covid‐19, including national and local restrictions, restricted the mode of training delivery. Project staff suggested that moving forward, online/self‐directed, and interactive training should be combined to support mobiliser learning and provide mobilisers with scenario‐based activities.

As a requirement of the programme, mobilisers were asked to complete the log book forms documenting interactions with peer beneficiaries, to support data gathering to enable AMAP staff to quantify the impact of the project. Some mobilisers were able to meet this requirement, documenting every mobilisation with a member of the public. Others however, felt it was burdensome. Participants suggested a less complicated log book form or a digital‐based record (such as a voice note that could be recorded quickly after an interaction) may be more acceptable to volunteers. Mobilisers were also required to ask peer beneficiaries if they were willing to complete a feedback form. Given the personal nature of the discussions, mobilisers reported they were hesitant to request peer beneficiaries to complete a feedback form‐as they felt this may be seen as intrusive.

Participants reflected on their learning as a result of engaging in the project and reported significant benefit in taking part in the pilot project, including developing their own PrEP‐related knowledge, while also developing interpersonal skills such as communication and confidence with public speaking.

Most mobilisers made use of the merchandise. Some carried a tote bag to prompt conversation, whilst others posted photos of themselves wearing merchandise via social media. Very few participants did not use the merchandise, and this was often for personal reasons ‐ such as not being public about their sexuality.

### 4.5 Mobilisation activity

As a result of their volunteering and conversations they had with peer beneficiaries, participants recognised the need for ongoing conversations around PrEP in their communities and in less often engaged groups. Many participants reported an ongoing need for improved awareness of PrEP, particularly around the practicalities of using PrEP and accessing PrEP through the NHS.

During the period of the pilot programme, many mobilisers were impacted by the Covid‐19 pandemic. Consequently, many of the face‐to‐face activities that had been planned by the mobilisers were shifted to online delivery, where possible. For some participants this was extremely challenging. Some participants were not public about their sexuality and could not utilise social media to discuss PrEP. Other participants were generally not comfortable with using social media and preferred having conversations with people face‐to‐face. However, other participants enjoyed the opportunity to host online events, or to use social media to engage people in conversations around PrEP. Imposter syndrome was reported by a few participants as they did not feel they had detailed clinical knowledge about PrEP to answer all the questions raised in conversations, but they explained that the AMAP training had helped them to overcome this. Overall, participants reported enjoying the autonomy of running independent health promotion projects whilst accessing the support and merchandise from the AMAP staff.

### 4.6 Support and social networking

A strong desire for networking/community/social elements to be part of the project was commonly expressed by participants. They described their participation in the project as being part of a community and contributing to community health. Participants suggested the creation of a What's App group to enable people to communicate and to organise social events and provide ongoing support to each other would be beneficial. Mobilisers did not always seem aware of the drop‐in support sessions and mentorship that was available to them, and whilst some participants did make use of the mentorship support, many forgot that they had a mentor, or how to contact them.

## 5 DISCUSSION

Our paper provides the first formal evaluation (following on from PrEPster's MobPrESH programme)^20^ of an England‐wide peer‐to‐peer HIV PrEP knowledge diffusion model. Our findings demonstrate that such models are highly acceptable to volunteer peer‐leaders (mobilisers) and can increase HIV PrEP outreach to significant numbers of people, who do not currently know about, or know how to use PrEP.

### 5.1 Attrition

The project experienced a significant dropout rate between initial project sign‐up for online training and the training event (pre‐training attrition), with 58 out of 138 people (42%) not attending. However, all participants who signed up for face‐to‐face training attended. Out of the 96 participants who completed training and volunteered, 8 people (8%) later withdrew (post‐training attrition) from the project. In total, out of the 154 initial applications to volunteer, 88 continued involvement in the pilot project until the end of the programme, providing a total attrition rate of 43%. We therefore suggest that similar projects consider recruiting more participants than initially required to allow for potential drop outs, to ensure sufficient volunteers are engaged, to meet long‐term project aims.^25^

### 5.2 Impact

Active mobilisers reported a high level of motivation to volunteer in peer‐led PrEP information programmes and were often motivated to do so by personal experience. Mobilisers were also motivated to continue to mobilise independently, once AMAP had completed. Despite initial disparity between registering interest in volunteering and completing induction training (pre‐training attrition), the post‐training attrition rate was low (8%). Mobilisers who completed training and remained engaged in the project were highly impactful. Throughout the duration of the 5‐month project mobilisers engaged their peers in 11,889 conversations about PrEP through individual conversations, group conversation, workplace educational events and online spaces. 889 conversations were in person and 11,000 conversations took place via social media.

Log form data demonstrates that 80 beneficiaries (24% of one‐to‐one conversations) reported their intention to start or restart PrEP following the AMAP intervention. Conversely, none of the log form data for online group conversations, online training events/forums, or social media posts reported this outcome. We suggest that this is likely due to the difference in the style of intervention between one‐to‐one and online interactions, and therefore the ability for mobilisers to gather in‐depth information about beneficiaries intentions relating to PrEP following an online interaction. Our data demonstrates the importance of one‐to‐one conversations which are more personal but with a smaller reach, and online conversations that whilst less interactive, may have the potential to increase PrEP awareness more widely. This is demonstrated in log form data which records one mobilser's social media post reaching 35,000 views.

### 5.3 Project delivery

AMAP training was designed to be ‘top‐level’ and of short duration, to enable busy people to engage. The training was subject to continuous review and development. Evaluation participants suggested that more scenario‐based exercises may be useful in future training sessions. Project staff suggested that online/self‐directed training may be a viable option for future projects. This would release staff from hosting multiple training events and allow recruits to undertake training at a time and speed that would be suitable for them. Associated costs with developing a training platform would need to be considered. However, some elements of group training should be retained to enable volunteers to develop social connections, which they valued.

Participants discussed their perspectives on the mobiliser log book forms, and peer beneficiary feedback forms which were used by project staff to analyse project impact. Many felt that the log book forms were burdensome. Overall, mobilisers felt that asking peer beneficiaries, to whom they had provided PrEP information, to provide feedback, was intrusive. This was particularly true in communities where people may feel the need to protect their identities.

The need for ongoing social support, and the development of ongoing networking opportunities was identified by mobilisers. Peer‐led PrEP diffusion models should consider the need for social networking outside of the formal project limits. However, project leaders should consider how such networks would be managed to ensure participant safety, and monitored to ensure evidence‐based and up‐to‐date PrEP information might be shared across groups by others not trained by the project. Supporting the development of a mobiliser social group enables knowledge sharing, and modelling of behaviours across wider interlinked external networks and communities which further supports the underpinning peer‐to‐peer diffusion aims of this project and aligns with the principles of Diffusion of Innovation Theory^21^ and Social Network Theory.^22^

### 5.4 Implications

Previous studies have reported inequity of access to PrEP, often through disparity in education and awareness about PrEP, sometimes due to limited discussion in social groups, marginalisation, poor representation, or insufficient targeting of groups such as Asian people,^30^ Black people,^30^ transgender people,^31^ and cisgender women.^32^ In our pilot programme Asian people represented 22% of the ethnicity demographic, and Black people (including Black African or Black Caribbean) represented 15% of the ethnicity demographic, transgender people represented 15% of the gender demographic, and females represented a further 15% of the gender demographic of beneficiaries who received an intervention. This demonstrates that peer‐to‐peer‐based diffusion models can go some way to addressing concerns about inequity of access to PrEP in some groups who are less likely to know about PrEP.

The National Institute for Health and Care Excellence: Reducing Sexually Transmitted Infections (NG221) guideline,^33^ highlights that young people aged 16–24 years are less likely to know about PrEP. Additionally, existing studies demonstrate that knowledge about how to use PrEP has been shown to be particularly poor in young people.^34^ In our pilot programme, people aged 21–29 years represented 50% of the age demograhic of beneficiaries, however people under 20 years represented only 5% of the age demographic of beneficiaries. We therefore suggest that future work should be focused on trialling the peer‐to‐peer diffusion method for young people aged 16–20 years old.

### 5.5 Study limitations

Focus group mobiliser participants repoted that completion of log forms to enable ongoing project impacts analysis was ‘burdensome.’ As such, it is possible that mobilisers may have carried out interventions that are not recorded in our impact data.

Additionally, the log book required mobilisers to report the demographic characteristics of people they spoke to, where it was possible to do so. The log book forms intentionally did not use drop‐down menus for mobilisers to complete to enable self‐identification, meaning that providing personal demographic data was sometimes challenging. Mobilisers did not usually report information about gender assigned at birth and ethnicity in log form data, resulting in incomplete demographic data. It is also likely that in some instances mobilisers may have made assumptions about gender or ethnicity based on conservations, incomplete knowledge, or the settings where an individual was encountered.

Our focus group mobiliser study sample included men who have sex with men and transgender men, including racially minoritised people, and people from diverse cultural, and geographical areas. However, our sample does not include cisgender women or transgender women who had been enrolled as mobilisers, so their views are not represented. Whilst the researchers made significant attempts to ensure equal representation of key demographics across participants, we acknowledge that some perspectives may have not been fully represented in our data.

Furthermore, the authors acknowledge the potential risk of self‐selection bias and desirability bias in our qualitative data. Further studies are needed to support our findings and explore further research highlighted in this paper before policy and practice recommendations can be supported.

## 6 CONCLUSION

This evaluation aimed to evaluate the first England‐wide national peer‐to‐peer based diffusion model to disseminate information about PrEP. The evaluation demonstrated volunteers could be recruited, trained and mobilised to disseminate information about accessing PrEP in England, using a flexible and blended approach, to people who are not typically included in public health campaigns. Further work could use similar models to continue to target groups which have been shown to have poor access to information about PrEP.

## AUTHOR CONTRIBUTIONS

Will Nutland, Marc Thompson, and Phil Samba were involved in the AMAP project conception, design, and implementation. Jonny Edwards and Sara Paparini were involved in the project evaluation conception and design, data collection, analysis, and interpretation of the data. Jonny Edwards drafted the original manuscript. Will Nutland and Sara Paparini critically revised the manuscript for intellectual content. Jonny Edwards, Sara Paparini, and Will Nutland approved the final version to be published. All authors agree to be accountable for all aspects of the work.

## CONFLICT OF INTEREST STATEMENT

The authors declare no conflicts of interest.

## Data Availability

The data that support the findings of this study are available from the corresponding author upon reasonable request.

## APPENDIX A ENROLLED MOBILISER FOCUS GROUP QUESTIONS

- Why did you take part in this project?
- How did/does the programme, once started, compare to what you expected in the beginning?
- How have you been getting on?
- How do you feel about the materials such as the log book forms?
- Can you think of 2–3 things you feel you have learnt by taking part in this programme?
- What are your experiences of the follow up and support that you have received from the team after training?
- If the programme were to continue, longer than initially planned (March 2022), what do you think you would want or need from PrEPster (or the project), on a long‐term basis, to keep you involved and motivated?

## APPENDIX B STAFF FOCUS GROUP QUESTIONS

- What were your initial hopes for the project?
- Did you experience any issues at the beginning of the project?
- Tell me about the uptake?
- Tell me about the training sessions?
- What are your views about the volunteers coming forward for training (motivations to volunteer, experiences of AMAP, and understanding of PrEP before training)?
- Were there any challenges or stand out successes?
- What would you have done differently?
- How do you feel the project is changing/adapting?
- Where do you see this project going in the future?
